# Applications of 3D Printing and Virtual Modeling in the Assessment of Visceral and Renal Artery Aneurysms

**DOI:** 10.3390/jcm14248915

**Published:** 2025-12-17

**Authors:** Daniel Grzegorz Soliński, Hanna Wiewióra, Wacław Kuczmik, Maciej Wiewióra

**Affiliations:** 1 Regional Specialist Hospital in Wroclaw, Research and Development Center, 51-124 Wroclaw, Poland; 2 SSA Vertex, Division of Anatomy, Department of Human Morphology and Embryology, Wroclaw Medical University, 50-368 Wroclaw, Poland; 3 Department of General Surgery, Vascular Surgery, Angiology and Phlebology, Faculty of Medical Sciences in Katowice, Medical University of Silesia, 40-635 Katowice, Poland

**Keywords:** 3D printing, virtual modeling, visceral artery aneurysms, renal artery aneurysms, interventional radiology, vascular surgery

## Abstract

**Background/Objectives**: The possibilities of endovascular and minimally invasive treatment of visceral and renal artery aneurysms require precise procedure planning. Accurate visualization of vascular pathologies is crucial in this regard. Expanding diagnostic imaging with real 3D models is one of these methods. The objective of our study was to evaluate the utility of 3D printing and virtual 3D models in visualizing visceral and renal artery aneurysms. **Methods**: A group of 30 patients with true aneurysms of the visceral and renal arteries was selected based on computed tomography angiography (CTA). Aneurysm morphology, diameters, arterial diameters, and anatomical vessel variants were analyzed. Imaging data were processed and 3D-printed using Fused Filament Fabrication (FFF) technology. The resulting 3D models were measured, and dimensional deviations were compared to radiological images. **Results**: The cohort included 51 aneurysms across arteries supplying abdominal organs, with splenic artery aneurysms (49%) and renal artery aneurysms (25.5%) predominating. Half of the patient group had multiple aneurysms, and 36.7% exhibited anatomical arterial variants. Forty-three 3D models of visceral and renal artery aneurysms were generated, accurately depicting isolated vascular pathologies and the course of visceral arteries in regions of interest. Measurement analysis confirmed that the 3D-printed models showed a mean dimensional deviation of 0.24 mm compared to radiological images. **Conclusions**: 3D-printed and virtual models enhance the analysis of vascular pathologies, significantly improving the assessment of pathological changes and visualization of anatomical details, especially in hilar aneurysms and aneurysm branches.

## 1. Introduction

Three-dimensional (3D) printing is increasingly incorporated into medical practice, notably for anatomical assessment, preoperative planning, intraoperative navigation, and surgical training [1]. Its value is especially evident when precise visualization of topography, geometry, and anatomical variations is required, complementing computed tomography and magnetic resonance imaging. Three-dimensional printing also shows significant potential in medical education, from foundational anatomy instruction to advanced simulations integrating virtual reality with 3D-printed models for procedural planning and training [2,3]. The visceral and renal arteries, which supply the abdominal organs and kidneys, exhibit considerable individual variability and may develop pathological changes, such as true aneurysms, which are challenging to visualize. These arteries display a wide range of anatomical variations in their origin, course, and relationship to surrounding structures, conspicuously in the branches of the celiac trunk and renal arteries, due to their complex geometry, numerous connections, and diverse configurations. Depending on their location and proximity to adjacent organs, visceral and renal aneurysms may be poorly visualized on ultrasound examination. Their nonspecific clinical presentation further complicates detection and diagnosis, which currently relies primarily on contrast-enhanced CT imaging. Although true visceral artery aneurysms (VAAs) and renal artery aneurysms (RAAs) are relatively rare, they pose a significant risk of severe complications, including potentially life-threatening rupture. The occurrence of VAAs varies among populations, reflecting their heterogeneity. This study aimed to assess the effectiveness of 3D printing and virtual 3D models in the visualization of visceral and renal artery aneurysms.

## 2. Materials and Methods

**Figure 1:**
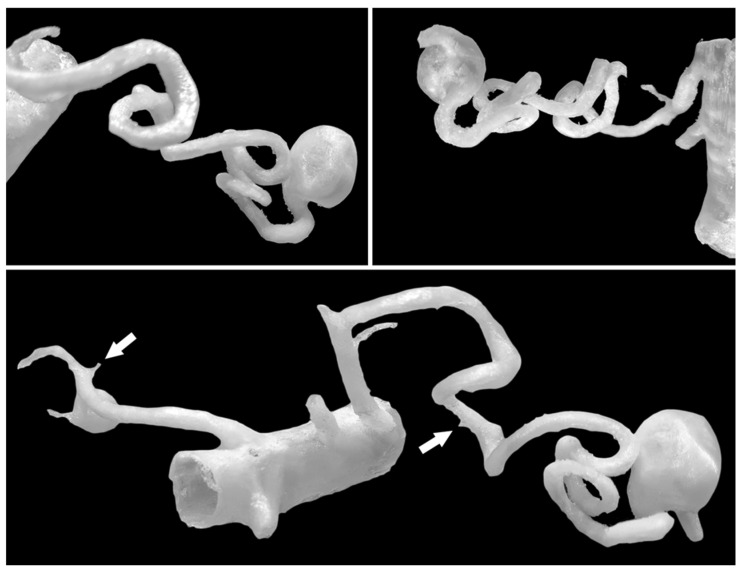
Spatial 3D models enable the creation of complex and tortuous arterial geometries. A single model can contain aneurysms in various regions—in this case, the SAA and RRAA. The origins of small arteries (<1 mm) are marked with white arrows, but their further course is cropped out as it is not important for the model and could unnecessarily complicate the printing process.

**Figure 2:**
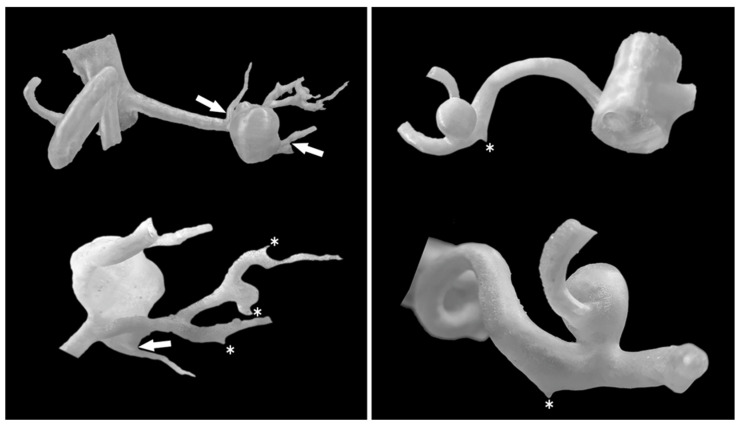
RAA models from various perspectives. Three-dimensional aneurysm models allow precise evaluation of the aneurysm sac positions, their morphology, and the arteries supplying and branching from them. Structures that are closely attached can be complex to separate in the model, but their apparent separation is preserved (white arrows). Asterisks mark the origins of small arteries. The segmental renal arteries in the model on the left have a minimum diameter of 1.29 mm.

A retrospective, single-center cohort of 30 patients with true aneurysms of the visceral and renal arteries, diagnosed via contrast-enhanced abdominal computed tomography angiography (CTA), was analyzed. The inclusion criterion was adequate vessel contrast on CTA imaging. Pseudoaneurysms and post-rupture aneurysms were excluded. No size-based exclusion criteria were applied, allowing evaluation of aneurysms across various sizes, morphologies, and disease stages. Subsequently performed and consulted imaging examinations were classified based on the diagnostic criterion, without other criteria for further management, without selection based on subsequent clinical decisions, and in order to minimize the impact of including aneurysms in the cohort based on their location, morphology, or vascular anatomical variants. Due to the retrospective nature of the group, which included aneurysms of various dimensions and morphologies, including those eligible for follow-up only, to assess the usefulness of 3D models in mapping imaging data, further clinical management was not considered in this analysis and did not affect the cohort. CTA images were reviewed using a radiology workstation [OsiriX MD software, version 14.x, Pixmeo SARL, Bernex, Switzerland, https://www.osirix-viewer.com/] by a specialist in radiology and diagnostic imaging, an interventional radiologist, who was also responsible for the further 3D printing process. Aneurysm sac diameters were measured at their maximum, along with arterial dimensions proximal and distal to the sacs. Vascular anatomical variants were assessed. Virtual models of isolated aneurysms and segments of supplying and draining arteries were generated from DICOM data using 3D Slicer software [3D Slicer, version 5.x, https://www.slicer.org/]. During the segmentation process, thresholds and postprocessing were individually selected based on the quality of the CTA output data, minimizing the impact on the virtual model to avoid possible distortion of the model’s geometry. These models were 3D printed using TPU95A material on Ultimaker 2+ (Ultimaker B.V., Zaltbommel, The Netherlands) or Original Prusa i3 MK3S+ (Prusa Research a.s., Prague, Czech Republic) printers. Forty-three 3D-printed models of visceral and renal artery aneurysms were fabricated, featuring hollow lumens of aneurysm sacs and main vessels. The 3D models were measured using an electronic caliper [Mitutoyo 500-196-30, Mitutoyo Corporation, Kawasaki, Japan] at the maximum aneurysm sac diameter, with each measurement repeated three times and averaged. Dimensional deviations were compared with radiological measurements. To maintain external dimensions and sac geometry, model walls were designed to narrow the internal lumen. Generating walls external to the contrast-enhanced vessel lumen would have introduced dimensional deviations proportional to wall thickness. When the vessel lumen was smaller than the specified wall thickness, it was printed solid, rendering the smallest branches non-hollow. Closely spaced arterial origins from aneurysm sacs or vessels aligned along the sacs were occasionally inseparable in virtual models. Adjacent vessels with significant curvature could create the false appearance of anastomoses or aneurysm sacs in virtual reconstructions. In cases of suboptimal CTA imaging, simultaneous contrast enhancement of veins and arteries within the same threshold range necessitated manual removal of extraneous elements from the model. To accurately represent sac wall structure and dimensions, mural thrombi and calcifications were manually incorporated into the model walls as needed. Tiny arterial branches were marked at their origins to minimize printing failures [Figure 1 and Figure 2].

## 3. Results

**Figure 3:**
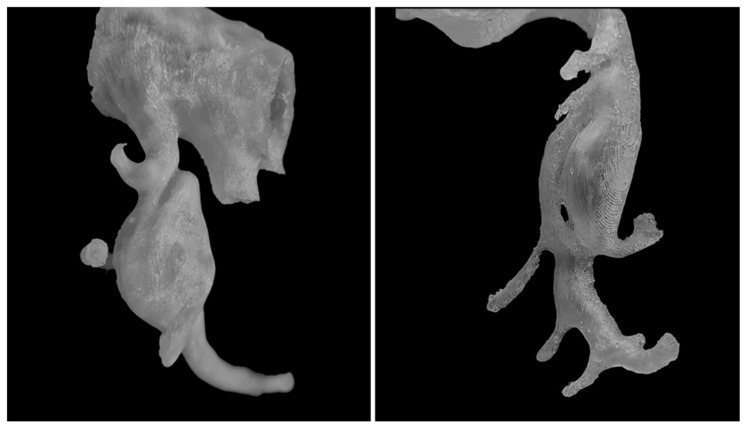
Arterial dissections. In 3D printed models, it is possible to visualize various vascular variants and pathologies, including dissections (on the (**left**)—dissection within CTAa) and visualization of dissection channels and arteries branching from them (on the (**right**)—SMA model).

**Figure 4:**
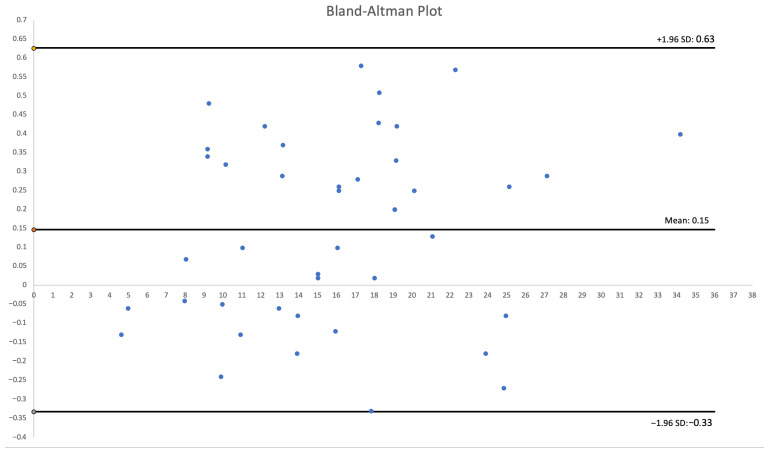
Bland–Altman Plot.

The cohort comprised 30 patients. There were 17 women (56.7%) and 13 men (43.3%) with a total of 51 visceral and renal artery aneurysms. The mean age was 56 years (range: 30–82 years), with a similar age distribution between genders. Half of the cohort (15 patients) had multiple aneurysms. Splenic artery aneurysms (SAAs) were the most prevalent, affecting 53.3% of patients and accounting for 25 of the total aneurysms (49%), predominantly in women. We found 20 SAAs in 12 females: 70.6% of female patients and 80% of all SAAs. SAAs were most commonly located at the splenic hilum (15 cases; 60%) or the distal segment of the splenic artery (5 cases; 20%). Morphologically, SAAs exhibited wide necks, wall calcifications, and mural thrombi, alongside tortuous arterial courses [Figure 1]. The mean diameter of the SA aneurysm sac was 14.32 mm (range: 3–25 mm). Renal artery aneurysms (RAAs) were the second most common type observed, affecting 11 patients (36.6%) and comprising 13 aneurysms, which accounted for 25.5% of all aneurysms. Of the 13 RAAs, 4 were located at the renal hilum and 4 at the main renal artery bifurcation (each 30.8% of RAAs), with branches arising directly from the aneurysm sacs, posing challenges for endovascular or surgical repair [Figure 2]. The mean RAA diameter was 14.6 mm (range: 5–27 mm). Other aneurysms included superior pancreaticoduodenal artery (SPDA) and inferior pancreaticoduodenal artery (IPDA) aneurysms in 4 patients (13.3%) accounting for 7.8% of all aneurysms, celiac trunk aneurysms (CTAn) in 4 patients (13.3%) accounting for 7.8% of all aneurysms, hepatic artery aneurysms (HAA) in 2 patients (6.7%) accounting for 3.9% of all aneurysms, and one aneurysm each of the superior mesenteric artery (SMA), left gastric artery (LGA), and left gastroepiploic artery (LGEA) aneurysms in 3 patients (10% of patients; each representing 2% of all aneurysms). Additional vascular pathologies included dissections in 4 patients: one extending from the aorta to the common iliac artery, two involving the celiac trunk, and one affecting the superior mesenteric artery (SMA) [Figure 3]. One patient had an abdominal aortic aneurysm treated with a stent graft, while 2 cases each of splenic artery occlusion and segmental celiac trunk occlusion were noted. The mean maximum diameter of aneurysm sacs was 14.78 mm, ranging from 3 mm (smallest SAA) to 34 mm (largest IPDA aneurysm). The mean diameter of arteries proximal to the aneurysm sacs was 4.4 mm. Distal arteries branching from sacs at vessel bifurcations or narrow segmental arteries were not measured. For measurable distal arteries, the mean diameter was 4.52 mm, showing no significant difference from proximal vessels. Anatomical variants were observed in 11 patients (36.7%). The most prevalent in RA, including high renal artery (RA) bifurcation in 3 patients (10%), accessory lower pole RA in 2 patients (6.7%), accessory upper pole RA in 2 patients (6.7%), accessory left RA arising from the abdominal aorta (AA) in 1 patient (3.3%). Others less frequent anatomical variation included right hepatic artery (RHA) originating from the SMA in 3 patients (10%), left hepatic artery (LHA) from the celiac trunk in 1 patient (3.3%), gastroduodenal artery (GDA) from the celiac trunk in 1 patient (3.3%), isolated left gastric artery (LGA) origin from the AA in 2 patients (6.6%), and common hepatic artery (CHA) from the SMA in 1 patient (3.3%). Three-dimensional printing time varied depending on the geometric complexity of the models and the associated support material, anatomical extent, and number of aneurysms, ranging from 2 h 5 min to 22 h 15 min per print. The mean diameter of the 3D-printed aneurysm sac was 16.02 mm. Dimensional deviations in the 3D-printed models ranged from 0.02 to 0.58 mm (mean: 0.24 mm, SD: 0.15 mm). Relative to CTA measurements, 3D-printed models showed mostly an overestimation of sac dimensions. Additionally, a Bland–Altman statistical analysis was performed and shown in the plot [Figure 4]. However, most discrepancies, up to 90% of printed models, were less than 0.5 mm, deemed clinically insignificant and not affecting geometric assessment (Table 1).

## 4. Discussion

VAAs and RAAs pose a clinical challenge due to their treatment modalities. The anatomy, anatomical variants of the arteries, and their visualization play a critical role in selecting the treatment method, endovascular tools, and procedural outcomes. The morphology and precise visualization of aneurysm sacs located at the hilum or with emanating branches are of significant importance. We found that the most common visceral aneurysm is the SAA, predominantly occurring in women, which is consistent with the literature [4]. The second most common aneurysm was the RAA. The study revealed significant anatomical variability in visceral arteries, and thus in potential endovascular access for aneurysm treatment. Three-dimensional printing effectively visualized the modeled arterial segments, including the aneurysm pathology, particularly its branches, which is crucial for planning minimally invasive treatment. Additionally, a very high morphometric accuracy was observed compared to CTA imaging. Visceral arteries are the main branches that arise from the abdominal aorta and distribute blood to the gastrointestinal tract. Anatomically, the celiac trunk has three branches: the common hepatic artery, the left gastric artery, and the splenic artery. A literature review on the anatomical variations in the celiac trunk, based on imaging studies or cadaver examinations, indicates that the typical trifurcation pattern is found in 62–90% of cases [5,6]. The most common variation is a bifurcation of the celiac trunk, which occurs in about 8% to 25% of cases. Bifurcation can give rise to a hepatosplenic trunk, where the left gastric artery arises from the abdominal aorta, occurring in 3.3% to 8.3% of cases. In our study, 6.6% of patients had this variant. Other variations in bifurcation include the hepatomesenteric trunk and the splenogastric trunk, where the common hepatic artery arises from the superior mesenteric artery with frequencies of 1.9% to 6.6% and 1.3% to 3.4%, respectively. We found hepatomesenteric variant with frequencies of 3.3%. Importantly, in cases of anatomical variations, the diameter of the arteries tends to be smaller than in cases with typical anatomy. This information can be useful for detailed and accurate radiological assessment during treatment planning, especially for VAAs. An anatomical variation where the splenic artery arises from the superior mesenteric artery is rare (0.7–1%). However, it can pose significant challenges in treatment, particularly when dealing with a splenic artery aneurysm [7]. The splenic artery can exhibit an unpredictable course and variation. A study on 320 cadavers assessed the origin, course, and terminal branches of the splenic artery, along with their division before entering the hilum of the spleen [8]. The splenic artery most commonly originates from the celiac trunk (up to 96.6%), from the abdominal aorta (up to 8.1%), or the common hepatic artery or the superior mesenteric artery (up to 1.3%). Most variations involved the terminal branches of the splenic artery after the division of the main trunk, while typical bifurcation into two branches occurred in 63% of cases. Moreover, a previously undocumented variation was discovered: the proximal part of the splenic artery wrapped around the neck of the pancreas and embedded itself in the pancreatic substance before giving rise to four branches outside of the pancreas. The greatest obstacles to radiological assessment and endovascular procedures are the highly variable terminal branches of the splenic artery and the regions of the splenic hilum, with a variation frequency ranging from 3% to 27% [9]. Similar to the splenic artery, the renal artery also exhibits anatomical variations, importantly in the case of hilar arteries [10]. The most common variations are accessory renal arteries (ARA), occurring in 20–62.2% of cases. ARAs can occur in variants ranging from one to three, either unilaterally or bilaterally. Accessory renal arteries most often occur at the hilum (up to 72.6%) and are significantly more frequent than polar accessory renal arteries. A common variant is the presence of additional polar renal arteries, occurring in 13.4% of our cohort. Their occlusion during surgery frequently leads to ischemia of part of the renal parenchyma [11]. Renal arteries can also undergo early division in the initial segment of the artery (up to 8.4%). In our group, the result was 10%. The course of the main renal artery (RA) anterior to the inferior vena cava is rare [12,13]. Specific variants of the renal artery originating even from the thoracic aorta have also been reported [14]. The authors emphasize that awareness of various anatomical variations before and during the planning of kidney surgeries or interventional procedures can be extremely beneficial, as it helps prevent potential complications. This knowledge is vital in surgical planning, especially for endovascular procedures related to the visceral blood supply, since curing pathologies requires minimizing organ ischemia. Closure of vessels branching from RAA sacs poses a risk of renal ischemia, which has greater implications for organ function compared to splenic ischemia from SAAs, where the impact on overall health and prognosis is less severe. The presence of various anatomical variations can have crucial clinical significance [15]. Pancreaticoduodenal artery (PDA) aneurysms posed significant challenges for assessment and visualization on CTA due to the tortuous arterial course, segmental dilatations, and proximity to adjacent vascular and organ structures in the abdominal cavity. Images from CTA scans allow for the isolation of contrast-enhanced arteries and the generation of virtual three-dimensional models. Despite the delineation of anatomical structures, these images remain two-dimensional with limited perceptual capabilities, as they do not engage the observer’s stereoscopic vision. Therefore, as noted in previous studies, 3D-printed models offer advantages over 2D images. Binocular vision, combined with the processing of somatosensory information and the manipulation of a physical object with hands, enhances stereognosis in operators. Additionally, the deformability of models can improve the understanding of vascular anatomy [16]. There is also an increasing focus on integrating 3D printed models with mixed reality as complementary methods that enable advanced training for operators. Three-dimensional models are supplemented with virtual elements that are difficult to print physically, along with concepts of operator interaction with the physical model, which can be helpful in neurosurgery for advanced brain tumor surgery training models [17]. Incorporating AI into image segmentation can significantly simplify and speed up the process, as seen in a web-based application that converts MRI scans into 3D-printable models without requiring software installation [18]. The use of virtual reality in cases requiring in-depth analysis is being considered as an alternative to 3D printing in acute, urgent cases [19]. Medical students and residents during their training achieved better outcomes in cases of learning and surgical training based on 3D-printed models [20]. The use of 3D printing in the management of visceral artery aneurysms remains a specialized field, with limited studies necessitating further investigation. Existing research highlights the high fidelity of physical models in replicating anatomical details, a finding corroborated by the present study. Key advantages include preoperative simulation of endovascular interventions, improved comprehension of the spatial relationships between the aneurysm sac and its supplying and branching arteries, and the potential to refine patient selection and surgical planning, favoring minimally invasive techniques. This approach proves valuable for aneurysms in complex anatomical locations, such as those near the renal or spleen hilum. Precise visualization using a 3D-printed model of a renal artery aneurysm, enabling enhanced depiction of the distal renal artery segments and segmental arteries, can facilitate decision-making for endovascular treatment, assessment of stent implantation feasibility, evaluation of the aneurysm neck, and analysis of the morphology of the embolized aneurysm sac [21]. In cases where computed tomography (CT) provides unambiguous results, 3D printing is not necessary. However, reliance solely on CTA imaging does not always provide sufficient clarity for decisions regarding minimally invasive procedures, and automatically generated virtual models may be prone to errors. In the presence of complex vascular geometry, anatomical variants, or overlapping anatomical structures, 3D printing enhances the ability to assess pathological changes visually. It facilitates decision-making regarding the selection of minimally invasive treatment. Three-dimensional models also facilitate communication among operators, physicians, and patients and can serve as intraoperative navigational support [22,23,24,25]. A randomized study investigating the impact of 3D printing on the outcomes of colorectal cancer treatment in patients undergoing hemicolectomy or anterior resection of the rectum has been conducted [26]. Patients were divided into two groups: one group where surgeons had access to CT and 3D-printed models of mesenteric vessels preoperatively and intraoperatively, and another group where access to 3D prints was granted only postoperatively. The study results indicated that the average duration of hemicolectomy and anterior resection of the rectum was significantly shorter, and the complication rate was lower in the group where surgeons utilized 3D-printed models. Additionally, surgeons who used 3D prints during the procedure reported that it was easier to identify vessels. Conversely, the second group encountered intraoperative complications, such as leaks or bowel necrosis, due to incorrect identification of the colon’s blood supply, particularly the absence of the left branch of the middle colic artery. Prior knowledge of this vascular variation, based on 3D prints, where branches of the left colic artery supply the left colic flexure and the distal part of the transverse colon, significantly altered the surgical approach. Similar results were obtained in another study, where the utilization of 3D printing before right hemicolectomy resulted in reduced operation time, decreased overall blood loss, and an increased number of lymph nodes removed [27].

### Technical and Clinical Limitations in Medical 3D Printing

The authors recognize several limitations in applying 3D printing for medical purposes. A critical factor is the expertise required in radiology and imaging of pathological conditions during model preparation, as these models may directly affect treatment planning. To ensure accuracy, 3D models should be developed in close collaboration with interventionalists and supervised by physicians specialized in radiology and diagnostic imaging. Artifacts in the input CTA data can block the generation of a 3D model, preventing 3D printing. From a clinical standpoint, dimensional discrepancies between CTA measurements and printed models of less than 1 mm are considered insignificant. This difference should not affect the choice of treatment method or approach, nor should it affect the geometric perception of the solid. However, these differences can result from model design choices, such as whether the model wall is defined external to, internal to, or centered on the contrast-enhanced vessel lumen. Such decisions should be customized based on the aneurysm sac size and the model’s intended purpose, particularly when testing surgical instruments. Additionally, the flexibility of the model walls may compromise measurement precision. In FFF technology, detailed and precise reproduction of small or closely adjacent vessels is challenging (separation may be impossible). The selection of materials and 3D printing technology can significantly impact the model’s utility, ranging from basic anatomical and morphological evaluation to preoperative tool testing. While high-quality models can confidently be produced with advanced 3D printers equipped with auto-calibration systems, not all printing methods produce models with consistent mechanical properties, depending on the materials employed. No 3D printing material has been tested and validated for medical applications that mimic the mechanical properties of blood vessels, which limits preoperative simulation and benchmark testing capabilities. The time required for 3D printing, its preparation, and subsequent processing for complex arterial geometries is lengthy, restricting its use to planned procedures and a limited number of patients. Despite good radiological and morphological assessment, endovascular management can be challenging and may require conventional surgery, for example, in renal hilum aneurysms. In such cases, 3D models can also be beneficial for planning conventional surgical procedures, as confirmed by previous case reports [28,29].

## 5. Conclusions

VAAs and RAAs are often not isolated lesions. Anatomical variants of arteries, diverse geometries of aneurysm sacs, and their locations, as identified in this study, can significantly complicate the process of endovascular treatment. Three-dimensional printing for advanced imaging may improve anatomical visualization and support minimally invasive techniques. Further development of these technologies is essential for their integration into routine medical practice.

## Figures and Tables

**Table 1:** Characteristics of the research group.

No.	Sex	Age	Aneurysm Location/Morphology	Diameter (mm) of the Aneurysm Sac (Angio-CT)	Diameter (mm) of the Aneurysm Sac (3D Printed Model)	Diameter Dimensional Deviation (mm)	Diameter (mm) of the Artery Before and After the Aneurysm Sac	Additional Information and Anatomical Vascular Variants
1.	F	58	SAA in the splenic hilum with 2 branches, complicated morphology of the aneurysm	20	20.25	0.25	4/3	N/A
2.	F	62	SAA, the tortuous course of the SA, wide neck of the aneurysm sac	18	17.67	0.33	4/5	The LGA originates from the AA superior to the CT. The RHA arises from the SMA. an additional LRA originates from the AA.
			SAA in the splenic hilum, branch of SA	3	N/A	N/A	N/A	
3.	F	42	SAA, the tortuous course of the SA, wide neck of the aneurysm sac	25	24.92	0.08	6/7	N/A
			SAA in the splenic hilum	4	N/A	N/A	N/A	
4.	F	55	SAA on the SA division with branches, in the splenic hilum	19	19.33	0.33	5/N/A	The RHA arises from the SMA.
			LGEAA with marginal calcifications and mural thrombi	13	13.37	0.37	4/N/A	
5.	M	52	SAA on SA trunk	25	24.73	0.27	7/7	Additional upper pole LRA. High division of the RRA.
6.	F	82	SAA, largest saccular aneurysm of the SA trunk with a wide neck	15	15.02	0.02	7/10	Well-defined marginal calcification of aneurysm sacs.
			SAA in the splenic hilum	16	16.26	0.26	3/N/A	
			SAA in the splenic hilum	9	9.34	0.34	3/3	
7.	F	65	SAA, largest aneurysm on the distal main SA trunk with mural thrombus up to 3 mm and calcifications	18	18.02	0.02	4/5	Additional lower pole LRA.
			SAA on SA trunk	5	4.94	0.06	N/A	
			SAA in the splenic hilum	11	11.1	0.1	3/N/A	
			SAA in the splenic hilum	13	13.29	0.29	4/4	
8.	F	49	SAA on the SA division in the splenic hilum	11	10.87	0.13	4/N/A	CHA originates from the SMA. Dissection of distal AA extending to the left CIA.
9.	F	46	SAA in the splenic hilum	16	15.88	0.12	4/N/A	N/A
10.	F	34	SAA, saccular aneurysm in the distal SA segment with two branching vessels	16	16.25	0.25	5/3	Isolated origin of the LGA from the aorta
11.	F	57	SAA, saccular aneurysm in the distal SA segment with marginal calcifications	19	19.42	0.42	4/4	Highly tortuous SA. RHA arises from the SMA. LHA is noted from the CT. GDA is noted from the CT.
			RAA of a RRA branch	10	9.95	0.05	N/A	
12.	M	65	SAA of one branch of the SA, in the splenic hilum, with a wide neck, well-calcified wall with small, mural thrombi	9	9.48	0.48	2.5/3	N/A
13.	F	30	SAA in the splenic hilum, distal SA segment, with several small branches	24	23.82	0.18	3.5/N/A	Additional upper pole RRA and LRA.
			SAA of an SA branch, in splenic hilum	6	N/A	N/A	N/A	
14.	M	71	SAA in the proximal SA segment with calcifications and small mural thrombi	21	21.13	0.13	4/4	Highly tortuous course of the distal SA segment.
15.	M	40	LRAA in the left renal hilum, complicated morphology	27	27.29	0.29	6/5	N/A
16.	M	68	RAA on the RRA division with 2 branches	17	17.28	0.28	4/N/A	N/A
17.	F	46	RRAA on a RA division with 3 branches	16	16.1	0.1	N/A	N/A
			RRAA of main RA trunk	9	9.36	0.36	5/N/A	
			CT dilatation with CT dissection	16	N/A	N/A	N/A	
18.	F	46	LRAA in the renal hilum at the main trunk division, 3 segmental branches	17	17.58	0.58	4/N/A	N/A
19.	M	33	LRAA on the main LRA trunk branch, 2 segmental branches	19	19.2	0.2	5/N/A	N/A
20.	M	74	RRAA on the main trunk branch	12	12.42	0.42	8/4	SA segmentally occluded, distally filled via collateral circulation.
			SAA, partially thrombosed, in distal SA	19	N/A	N/A	N/A	
21.	F	55	RRAA branch in renal hilum, with several small segmental arteries, aneurysm sac with mural thrombus and calcified atherosclerotic plaques	10	10.32	0.32	2/N/A	RRA with early division.
			LRAA on the main trunk branch, with single branch, in renal hilum	10	9.76	0.24	3/4	
22.	M	69	RRAA on the RA division, with 2 branches arising from the sac, calcified plaques and small mural thrombi	25	25.26	0.26	5/N/A	Post-stentoplasty of the RA—proximal stent end visible in the sac, extending to one branch; stent patent.
23.	M	44	HAA in the proximal HA segment constricting the HA	22	22.57	0.57	N/A	MALS
			Post-stenotic dilatation of the CT	10	N/A	N/A	N/A	
24.	F	74	Multiple dilatations of SPDA and IPDA arteries	18	18.43	0.43	N/A	N/A
			RRAA	5	N/A	N/A	N/A	
25.	M	71	Aneurysm near the IPDA origin from the SMA, with several jejunal arteries arising from the sac; multiple dilatations of SPDA and IPDA arteries	34	34.4	0.4	6/6	CT occluded at origin, subtotally narrowed distally, filled via collateral branches.
			SAA in the splenic hilum	8	N/A	N/A	N/A	
26.	F	55	Saccular IPDA aneurysm	14	13.92	0.08	4/2	CT occluded at origin, subtotally narrowed distally, filled via collateral branches.
			SAA in the splenic hilum, on SA division	8	8.07	0.07	4/3	
			CHAA	8	7.96	0.04	5/4	
			Segmental RAA	13	12.94	0.06	2/N/A	
27.	F	38	Aneurysm near the IPDA origin from the SMA; several dilatations of SPDA and IPDA arteries	19	19.2	0.2	4/5	Severe CT stenosis.
28.	M	67	Fusiform, dissecting SMA aneurysm	14	13.82	0.18	7/6	Post-treatment of AAA with stent graft.
			LGA aneurysm	4.7	4.57	0.13	2/2	
29.	M	71	Fusiform CT aneurysm	15	15.03	0.03	5/5	Additional lower pole RRA.
30.	M	62	Fusiform CT aneurysm proximal to the CT division with the LGA arising from the sac, short-segment CT dissection at the aneurysm neck	18	18.51	0.51	6/N/A	SA narrow in proximal third, distally occluded; post-splenectomy; high RRA division.

## Data Availability

The original contributions presented in this study are included in the article. Further inquiries can be directed to the corresponding author.

## Abbreviations

N/A	Not Applicable
3D printing	Three-Dimensional Printing
AA	Abdominal Aorta
CHA	Common Hepatic Artery
CHAA	Common Hepatic Artery Aneurysm
CIA	Common Iliac Artery
CT	Celiac Trunk
CTA	Computed Tomography Angiography
FFF	Fused Filament Fabrication
GDA	Gastroduodenal Artery
HAA	Hepatic Artery Aneurysm
HA	Hepatic Artery
IPDA	Inferior Pancreaticoduodenal Artery
LGA	Left Gastric Artery
LGEAA	Left Gastroepiploic Artery Aneurysm
LHA	Left Hepatic Artery
LRA	Left Renal Artery
MALS	Median Arcuate Ligament Syndrome
RA	Renal Artery
RAA	Renal Artery Aneurysm
RAAs	Renal Artery Aneurysms
RHA	Right Hepatic Artery
RRA	Right Renal Artery
RRAA	Right Renal Artery Aneurysm
SA	Splenic Artery
SAA	Splenic Artery Aneurysm
SMA	Superior Mesenteric Artery
SPDA	Superior Pancreaticoduodenal Artery
VAAs	Visceral Artery Aneurysms
